# Prevalence and factors associated with the correlation between malnutrition and pain in hemodialysis patients

**DOI:** 10.1038/s41598-024-65603-2

**Published:** 2024-06-27

**Authors:** Mohammad M. Jaber, Mazen A. Abdalla, Aya Mizher, Heba Hammoudi, Farah Hamed, Abrar Sholi, Adham AbuTaha, Mohannad Hassan, Sari Taha, Amer A. Koni, Muna Shakhshir, Sa’ed H. Zyoud

**Affiliations:** 1 Department of Medicine, College of Medicine and Health Sciences, An-Najah National University, Nablus, 44839 Palestine; 2 Department of Biomedical Sciences, Faculty of Medicine and Health Sciences, An-Najah National University, Nablus, 44839 Palestine; 3 Department of Pathology, An-Najah National University Hospital, Nablus, 44839 Palestine; 4 Department of Orthopedic Surgery, An-Najah National University Hospital, Nablus, 44839 Palestine; 5 Department of Nephrology, An-Najah National University Hospital, Nablus, 44839 Palestine; 6 An-Najah Global Health Institute, An-Najah National University, Nablus, 44839 Palestine; 7 Division of Clinical Pharmacy, Department of Hematology and Oncology, An-Najah National University Hospital, Nablus, 44839 Palestine; 8 Department of Clinical and Community Pharmacy, College of Medicine and Health Sciences, An-Najah National University, Nablus, 44839 Palestine; 9 Department of Nutrition, An-Najah National University Hospital, Nablus, 44839 Palestine; 10 Clinical Research Center, An-Najah National University Hospital, Nablus, 44839 Palestine

**Keywords:** Hemodialysis, Dialysis, Chronic kidney disease, Pain, Malnutrition, Diseases, Kidney diseases, Nutrition disorders

## Abstract

Malnutrition and pain are common in patients with chronic kidney disease who undergo hemodialysis. Although both pain and malnutrition are associated with increased morbidity and mortality, few studies have explored the correlation between pain and nutritional status. This study aimed to investigate the factors associated with pain intensity in patients undergoing hemodialysis, focusing on the risk of malnutrition. This was a cross-sectional study conducted at a regional dialysis center in a large tertiary hospital. Convenience sampling was used to recruit adult patients who had undergone hemodialysis for more than three months. An interviewer-administered questionnaire was used to gather sociodemographic and clinical data related to dialysis status, comorbidities, and body mass index (BMI). Pain severity and pain interference with functioning domains of the Brief Pain Index (BPI) were used to assess pain, and the malnutrition inflammation score (MIS) was used to assess nutritional status. Descriptive and inferential statistics were used to report the findings. The data were analyzed using the 25th version of the Statistical Package for the Social Sciences (IBM-SPSS) software. Of the final sample of 230 patients, 63.0% were males and 37.0% were females, with an average age of 58.3 years. Almost one-third of the participants had a BMI within the normal range (33.9%), and nearly one-third had a BMI within the underweight range (33.9%). Slightly more than half had a normal nutritional status or mild malnutrition (54.8%), while just under half had moderate or severe malnutrition (45.2%). The prevalence of pain was 47.0%. At the multivariate level, the severity of pain was associated with malnutrition (*p* < 0.001). Pain interference with function was associated with marital status (*p* = 0.045), number of comorbidities (*p* = 0.012), and malnutrition *(p* < 0.001). The MIS was positively correlated with both the severity of pain and the interference score. Pain and malnutrition were found to be prevalent in patients undergoing hemodialysis. Pain severity was associated with malnutrition, and pain interference was associated with malnutrition, marital status, and the number of comorbidities. Hemodialysis treatment should follow a patient-tailored approach that addresses pain, nutritional status, and associated chronic conditions. In addition, pain assessment and management should be included in the curriculum of nephrology training programs.

## Introduction

Malnutrition, in terms of undernutrition, is defined as the deficient intake or uptake of nutrients that can manifest as stunting, wasting, underweight status, or micronutrient deficiencies. Malnutrition is a global health problem whose burden disproportionately affects low- and middle-income countries (LMICs). In 2014, approximately 462 million people were estimated to be underweight worldwide^1^. Elderly patients are particularly vulnerable to diseases^2^, cognitive impairment^3^, frequent hospitalization^4^, poor oral health^5^, and age-related changes in body physiology, appetite, and taste^6–8^. Malnutrition leads to decreased muscle and fat mass, jeopardizing muscular, cardiac, and respiratory functions. Furthermore, malnutrition can lead to impaired immunity, poor wound healing, and nutrient malabsorption^9^.

Although the global burden of chronic kidney disease (CKD) did not change between 1990 and 2017, hemodialysis has become a more common treatment option. The age-standardized incidence of hemodialysis increased by 10.7% during the same period, and more than 2.5 million people received hemodialysis in 2017^10^. Malnutrition is common, particularly in patients undergoing hemodialysis, the prevalence of which ranges between 42.4 and 54.5%^11–13^. The serum albumin concentration and prealbumin concentration have been suggested to be indicators of nutritional status and to be predictors of morbidity and mortality in patients undergoing hemodialysis^14,15^. Malnutrition is also associated with cognitive decline^16^ and reduced quality of life^17,18^.

Pain is also a common complaint in patients undergoing hemodialysis. A systematic review by Brkovic et al. reported that the prevalence of pain among patients undergoing dialysis varies widely across settings but can reach 82% and 92% for acute and chronic pain, respectively^19^. In response to their pain, patients may have negative thoughts and emotions and adopt negative attitudes toward their treatment. Chronic pain can, for example, interfere with people’s participation in life and work activities^20^, drain cognitive capacity^21^, invoke hopelessness, and affect their will to live^22,23^. In addition, pain often worsens the experience of dialysis^24^ or dialysis-related procedures, such as cannulation^25^. Notably, pain is associated with a decrease in the glomerular filtration rate (GFR) and an increase in dialysis mortality in patients^26,27^.

The relationship between pain and nutritional status is bidirectional and complex. A study reported that hospitalized elderly patients with severe pain are nearly 1.4 times more likely to be at risk of malnutrition than patients without pain^28^. In another study conducted among elderly patients, chronic nonmalignant pain was significantly associated with poor appetite^29^. However, the mechanism and directionality of the pain-nutrition relationship remain unclear. In view of the potential association between pain and nutrition, improving nutritional status and dietary habits has been suggested as a treatment modality in pain management^30^.

Malnutrition and pain in patients undergoing hemodialysis have been extensively studied. In Palestine, multiple studies have indicated a high prevalence of malnutrition and pain in this patient population and revealed that various demographic, socioeconomic, and clinical factors are associated with both^11,17,31,32^. However, no global or local studies have explored the relationship between malnutrition risk and pain intensity in patients undergoing hemodialysis. This is especially crucial in light of the possible relationship between pain and malnutrition among other populations^30^. Exploring this relationship might contribute to improving hemodialysis care and establish the foundation for further research. This study aimed to investigate the demographic, socioeconomic, and clinical factors that influence pain intensity, with a focus on the risk of malnutrition.

## Methods

### Study design and settings

This was a single-center cross-sectional study conducted at An-Najah National University Hospital (NNUH) in Palestine from 2021 to 2022. The hospital has a major dialysis center whose catchment area is the northern governorates of the West Bank.

### Population and sampling

A total population of 350 patients received dialysis at NNUH in 2019 according to the annual health records published by the Palestinian Ministry of Health^33^. The *Raosoft* online sample size calculator was used to determine the minimum sample size. Using a desired confidence level of 95% and an accepted margin of error of 5%, the minimum sample size was estimated to be 184. A larger sample of the total population was obtained via convenience sampling. Patients who had been undergoing hemodialysis sessions for more than three months before the study and were older than 18 years were included. Patients were excluded if they had cognitive or mental limitations that could interfere with their autonomy and understanding of the study.

### Data collection: procedures, tools, and definitions of variables

#### Demographic, socioeconomic, and clinical data

The patients were invited to participate in the study and provided information using an interviewer-administered questionnaire, which was constructed and cross-checked for precision and clarity by professors and researchers with relevant experience. Data collection was performed during the dialysis sessions. The first part of the questionnaire included questions on demographic characteristics, including age, gender, height, weight, and socioeconomic characteristics, including residency (city, village, refugee camp), marital status (married, single), education level (did not receive formal education, primary level, high school-level, university-level), occupation (employed, unemployed), monthly income level (low-income level for an income < 2000 *NIS*; medium income level for an income ranging between 2000 and 5000 *NIS*; and high-income level for an income > 5000 *NIS*), and smoking status as a binary variable (smoker if a participant currently smokes and has smoked 100 cigarettes during a lifetime; and previously smoker and nonsmoker if otherwise)^34^.

The second part of the questionnaire included questions on clinical data, including the duration of dialysis (≤ 4 years, > 4 years); hours of dialysis per session (< 4 or ≥ 4); frequency of dialysis (2 sessions/week, 3 sessions/week, or ≥ 3 session/week); number of chronic diseases (< 4 or ≥ 4); number of medications used (< 4 or ≥ 4); history of kidney transplant (yes/no); and body mass index (BMI) classified into the following: underweight range, BMI < 18.5 kg/m^2^; normal weight range, BMI = 18.5–24.9 kg/m^2^; overweight range, BMI = 25.0–29.9 kg/m^2^l; and obese and morbid obese range, BMI > 30.0 kg/m^2^^35^.

#### Malnutrition-Inflammation Score (MIS)

Malnutrition status was assessed using the 10-component Malnutrition-Inflammation Score (MIS), which relies on collecting clinical data and conducting physical examinations and laboratory tests. The ten components are a medical history of disease; dry weight change; gastrointestinal symptoms; ability to function at work; physical examinations of muscle wasting (examined in temporal muscle, rib, clavicular, scapular, quadriceal, and knee); depleted fat stores (examined below eyes, chest, bicep, and triceps, and biceps); BMI (with an assigned score of 0 if BMI ≥ 20 kg/m^2^; 1 if BMI = 18–19.9 kg/m^2^; 2 if BMI = 16–17.99 kg/m^2^; and 3 if BMI < 16 kg/m^2^); and serum albumin and total iron binding capacity (TIBC). Each of the ten components is graded on a 4-point intensity scale ranging from 0 (normal) to 3 (severe). The final score was calculated by summing each component score and ranged from 0 to 30, with an MIS < 9 indicating no-to-mild malnutrition, 9–18 indicating moderate malnutrition, and > 18 indicating severe malnutrition^36,37^. The MIS has been shown to be a valid predictor of morbidity and mortality in patients undergoing dialysis^36,38^.

#### Brief Pain Inventory (BPI)

The Brief Pain Inventory (BPI) is a self-administered questionnaire that evaluates pain experience across four domains: pain severity, pain interference with functioning, pain location, and pain relief^39,40^. This study used the two domains of pain intensity and pain interference with functioning, each of which was reported separately as a continuous variable out of 10. First, the domain of severity of pain captures temporal pain variations by subjectively rating pain on a scale from 0 (as mildest) to 10 (as the most severe) using four elements: pain at its least and worst severity during the last 24 h, its average severity, and its current severity at the time of completing the questionnaire. Second, the pain interference domain with functioning assesses pain interference through seven life activities and is graded from 0 (noninterfering) to 10 (most interfering). The final scores for pain severity and dysfunctional dysfunction are reported as the arithmetic means of the individual item scores of pain severity and interference with functioning, respectively^41^. The pain intensity domain of the BPI has demonstrated acceptable to excellent internal consistency^42,43^, reliability^43–45^, and responsiveness to changes^43,46^ in nonmalignant conditions and has good reliability and validity in measuring pain in Palestine^18,47–51^. We obtained permission to utilize the officially validated Arabic version of the BPI short form provided by the MD Anderson Cancer Center.

### Data analysis

The data were analyzed using the 25th version of the Statistical Package for the Social Sciences (IBM-SPSS) software. The mean, range, and standard deviation are reported for normally distributed variables, such as age. Percentages and frequencies of categorical variables, including sociodemographic and clinical characteristics. The frequency, percentage, median, and mean rank were reported for continuous variables with a nonnormal distribution. The Shapiro‒Wilk test was used to assess the normality of the MIS and BPI scores. At the bivariate analysis level, the Mann–Whitney U test was used to analyze associations between pain and other variables in two categories, and the Kruskal–Wallis H test was used for variables in more than two categories. The MIS was defined as an ordinal variable at the bivariate level, as previously explained. In the multivariate analysis, a linear regression model was used to test the significance of the relationship between the BPI score and the variables that were found to be significant at the bivariate level. Based on the nonnormality assumption of variable distributions, the Spearman rank correlation method was used to examine the correlation between the BPI and MIS, both of which were defined as continuous variables. The significance level was determined with a *p* value less than 0.05.

### Ethics approval and consent to participate

The study received approval from the Institutional Review Board (IRB) of An-Najah National University, and the necessary permission documents were issued. Participants were given the freedom to accept or decline the invitation to participate voluntarily. The verbal informed consent of each participant who agreed to participate in the study was obtained, ensuring the confidentiality of their data. The IRB of An-Najah National University specifically approved the use of informed verbal consent due to the nature of the study, where participants participated only in interviews and clinical examinations without any potential harm, as long as their privacy was maintained. The authors affirm that all the methods adhere to the relevant guidelines and regulations.

## Results

### Demographic and clinical characteristics

Of the 280 patients approached, 23 declined to participate (8.2%), and 27 (9.6%) were excluded based on the exclusion criteria. Of the final sample of 230 patients, 145 (63.0%) were males, and 85 (37.0%) were females. The average age of the participants was 58.3 years (range: 18–85 years, *SD* ± 14.5), and 126 (84.8%) participants were aged older than 60 years. Most of the participants were married (77.4%), unemployed (84.8%), had a low income (64.3%), and had a primary education (46.1%). Most of the participants resided in cities (47.4%) or villages (42.2%), while a tiny minority lived in refugee camps (10.4%).

For BMI, 78 (33.9%) participants were within the normal range, and another 78 (33.9%) were within the overweight range. Less than one-third (27.0%) of the participants were obese or had morbid obesity, while only 12 (5.2%) participants were underweight. More than half had an MIS within the normal-to-mild malnutrition range (54.8%), while slightly less than half had moderate or severe malnutrition (45.2%). Most of the participants were nonsmokers or previous smokers (76.5%). Most of the participants reported having fewer than three chronic diseases (52.2%) and taking more than four medications per day (80.9%). Most had been receiving hemodialysis three times a week (92.6%), for less than 4 years (68.3%), and for less than four hours per session (91.3%) (Table 1).

**Table 1:** Association between pain severity score and other sociodemographic and clinical variables.

Variable		Frequency (%)	Mean rank	Median (Q1–Q3)	p value
		N = 230			
Age category (years)	18–44 years	41 (17.8)	104.23	0.0 (0.0–3.1)	0.154
	45–59 years	63 (27.4)	108.88	0.0 (0.0–3.3)	
	> 60	126 (84.8)	122.48	1.3 (0.0–4.6)	
Gender	Male	145 (63.0)	109.07	0.0 (0.0–3.3)	0.038*
	Female	85 (37.0)	126.47	1.3 (0.0–4.6)	
Marital status	Single, divorced, widow	52 (22.6)	129.72		0.057
	Married	178 (77.4)	111.35		
Household income level	Low-level	148 (64.3)	118.27	0.0 (0.0–4.5)	0.331
	Mid-level	71 (30.9)	113.62	0.0 (0.0–3.8)	
	High-level	11 (4.8)	90.36	0.0 (0.0–1.5)	
Residency	City	109 (47.4)	112.96	0.0 (0.0–4.1)	0.568
	Village	97 (42.2)	115.35	0.0 (0.0–4.0)	
	Camp	24 (10.4)	127.67	1.4 (0.0–5.2)	
Educational level	Did not received formal education	22 (9.6)	152.27	3.9 (0.6–5.3)	0.026*
	Primary	106 (46.1)	113.57	0.0 (0.0–4.3)	
	High school	55 (23.9)	112.61	0.0 (0.0–3.8)	
	University	47 (20.4)	106.03	0.0 (0.0–3.3)	
Occupation	Employed	35 (15.2)	107.11	0.0 (0.0–4.3)	0.380
	Unemployed	195 (84.8)	117.01	0.0 (0.0–4.3)	
BMI	Underweight	12 (5.2)	146.50	3.0 (0.0–6.3)	0.117
	Normal weight	78 (33.9)	114.88	0.0 (0.0–3.4)	
	Overweight	78 (33.9)	105.69	0.0 (0.0–3.2)	
	Obese & morbid obese	62 (27.0)	122.61	0.4 (0.0–4.8)	
Smoking status	Yes	54 (23.5)	110.13	0.0 (0.0–3.3)	0.462
	No or previously smoker	176 (76.5)	117.15	0.0 (0.0–4.5)	
MIS classification	No-to-mild	126 (54.8)	102.87	0.0 (0.0–2.8)	< 0.001*
	Moderate	99 (43.0)	126.77	2.3 (0.0–4.5)	
	Severe	5 (2.2)	210.80	6.3 (5.4–8.5)	
Dialysis years	≤ 4 years	157 (68.3)	111.77	0.0 (0.0–3.8)	0.177
	> 4 years	73 (31.7)	123.51	1.3 (0.0–5.1)	
Frequency of dialysis per week	≤ 2/week	14 (6.1)	148.29	3.9 (0.0–5.8)	0.117
	3/week	213 (92.6)	113.28	0.0 (0.0–4.1)	
	> 3/week	3 (1.3)	120.17	2.3 (1.1–2.6)	
Hours of dialysis per session	< 4 h	210 (91.3)	115.60	0.0 (0.0–4.3)	0.939
	≥ 4 h	20 (8.7)	114.50	0.0 (0.0–4.8)	
Number of chronic diseases	< 3 diseases	120 (52.2)	103.45	0.0 (0.0–3.0)	0.002*
	≥ 3 diseases	110 (47.8)	128.64	1.6 (0.0–4.8)	
Number of medications	< 4 drugs	44 (19.1)	112.32	0.0 (0.0–4.1)	0.702
	≥ 4 drugs	186 (80.9)	116.25	0.0 (0.0–4.3)	
History of kidney transplant	Yes	21 (9.1)	128.00	2.0 (0.0–3.8)	0.327
	No	209 (90.9)	114.24	0.0 (0.0–4.3)	

BMI body mass index; MIS: Malnutrition Inflammation Score; BPI: Brief Pain Inventory.

Significant values are in bold.

### Prevalence of chronic pain and malnutrition

The MIS and pain intensity scores were found to be nonnormally distributed based on the normal and detrended normal QQ plots and the Shapiro‒Wilk test results, with a *p* value < 0.001. The prevalence of pain, regardless of intensity, was 47.0%. The median BPI was 0.0 (Q1–Q3 = 0.0–4.3), the median pain interference score was 0.0 (Q1–Q3 = 0.0–5.2), and the median MIS was 8.0 (Q1–Q3 = 6.0–11.0).

### Factors associated with pain severity and interference

Bivariate analysis revealed that gender (*p* = 0.038), education level (*p* = 0.026), number of chronic diseases (*p* = 0.002) and MIS classification (*p* < 0.001) were significantly associated with the pain severity score (Table 1). The pain interference score was associated with gender (*p* = 0.011), marital status (*p* = 0.021), education level (*p* = 0.003), number of chronic diseases (*p* = 0.001), and MIS classification (*p* < 0.001) (Table 2). According to Spearman’s rank correlation, the MIS score was positively correlated with the pain severity score (*ρ* = 0.308, *p* < 0.001) and the pain interference score (*ρ* = 0.324, *p* < 0.001). The pain severity and interference scores were significantly positively correlated (*ρ* = 0.941, *p* < 0.001).

**Table 2:** Association between the pain interference score and other sociodemographic and clinical variables.

Variable		Frequency (%)	Mean rank	Median (Q1–Q3)	p value
		N = 230			
Age category (years)	18–44 years	41 (17.8)	104.45	0.0 (0.0–4.0)	0.108
	45–59 years	63 (27.4)	107.41	0.0 (0.0–4.0)	
	> 60	126 (84.8)	123.14	0.0 (0.0–6.0)	
Gender	Male	145 (63.0)	107.71	0.0 (0.0–4.0)	0.011*
	Female	85 (37.0)	128.79	2.1 (0.0–69)	
Marital status	Single, divorced, widow	52 (22.6)	132.65	3.1 (0.0–6.8)	0.021*
	Married	178 (77.4)	110.49	0.0 (0.0–4.1)	
Household income level	< 2000 NIS	148 (64.3)	119.13	0.0 (0.0–6.0)	0.339
	2000–5000 NIS	71 (30.9)	111.15	0.0 (0.0–4.0)	
	5000–10,000 NIS	11 (4.8)	94.82	0.0 (0.0–2.6)	
Residency	City	109 (47.4)	113.17	0.0 (0.0–4.6)	0.736
	Village	97 (42.2)	116.07	0.0 (0.0–5.7)	
	Camp	24 (10.4)	123.77	2.4 (0.0–5.9)	
Educational level	Uneducated	22 (9.6)	159.34	5.5 (1.9–7.3)	0.003*
	Primary	106 (46.1)	112.94	0.0 (0.0–4.9)	
	High school	55 (23.9)	114.55	0.0 (0.0–4.9)	
	University	47 (20.4)	101.86	0.0 (0.0–3.1)	
Occupation	Employed	35 (15.2)	102.97	0.0 (0.0–4.0)	0.186
	Unemployed	195 (84.8)	117.75	0.0 (0.0- 5.4)	
BMI	Underweight	12 (5.2)	150.83	4.6 (0.0–8.4)	0.092
	Normal weight	78 (33.9)	115.12	0.0 (0.0–4.9)	
	Overweight	78 (33.9)	106.06	0.0 (0.0–4.1)	
	Obese & morbid obese	62 (27.0)	121.02	0.0 (0.0–5.7)	
Smoking status	Yes	54 (23.5)	109.62	0.0 (0.0–4.1)	0.417
	No or previously smoker	176 (76.5)	117.30	0.0 (0.0–5.5)	
MIS classification	No-to-mild	126 (54.8)	101.60	0.0 (0.0–2.9)	< 0.001*
	Moderate	99 (43.0)	128.74	2.4 (0.0–6.7)	
	Severe	5 (2.2)	203.70	8.4 (5.2–8.9)	
Dialysis years	≤ 4 years	157 (68.3)	112.78	0.0 (0.0–5.0)	0.320
	> 4 years	73 (31.7)	121.35	1.9 (0.0–5.4)	
Frequency of dialysis per week	≤ 2/week	14 (6.1)	145.75	3.9 (0.0–7.1)	0.091
	3/week	213 (92.6)	113.02	0.0 (0.0–4.9)	
	> 3/week	3 (1.3)	150.33	5.4 (0.0–6.7)	
Hours of dialysis per session	< 4 h	210 (91.3)	116.16	0.0 (0.0–5.3)	0.594
	≥ 4 h	20 (8.7)	108.58	0.0 (0.0–4.6)	
Number of chronic diseases	< 3 diseases	120 (52.2)	102.48	0.0 (0.0–3.7)	0.001*
	≥ 3 diseases	110 (47.8)	129.71	2.5 (0.0–6.8)	
Number of medications	< 4 drugs	44 (19.1)	106.77	0.0 (0.0–3.9)	0.290
	≥ 4 drugs	186 (80.9)	117.56	0.0 (0.0–5.4)	
History of kidney transplant	Yes	21 (9.1)	126.00	2.9 (0.0–4.6)	0.407
	No	209 (90.9)	114.44	0.0 (0.0–5.3)	

BMI body mass index; MIS: Malnutrition Inflammation Score; BPI: Brief Pain Inventory.

Significant values are in bold.

### Multiple linear regression analysis

According to the linear regression model, only the association with the MIS classification (*p* < 0.001) remained significant for the pain severity score (Table 3). The results of the linear regression models for the pain interference score demonstrated a different pattern than that of the pain severity score, and marital status (*p* = 0.045), the number of chronic diseases (*p* = 0.012), and the classification of MIS (*p* < 0.001) retained significance (Table 3).

**Table 3:** Multivariate linear regression analysis of pain severity and interference with functioning scores.

Model	Significance (with pain severity score)	Significance (with pain interference score)
Constant	0.402	0.605
Gender	0.222	0.418
Education	0.496	0.183
Marital status	–	0.045*
Number of chronic diseases	0.066	0.012*
MIS classification	< 0.001*	< 0.001*

Dependent variable: pain severity measured by the Brief Pain Inventory (BPI) as a continuous variable.

MIS: Malnutrition Inflammation Score; BPI: Brief Pain Inventory.

*p value is below the threshold value for significance (0.05).

Significant values are in bold.

## Discussion

Hemodialysis, a renal replacement therapy, is an effective solution for treating renal failure. However, this solution comes at physical and psychological costs. Pain and malnutrition, particularly during dialysis, are common and are associated with increased morbidity and mortality. Given that little research has investigated the interaction between pain and nutritional status, this study focused on exploring the correlation between pain and the risk of malnutrition, in addition to the possible factors influencing pain in patients undergoing hemodialysis. The study showed that pain severity was associated with malnutrition, and pain interference with function was associated with malnutrition, marital status, and the number of comorbidities. The study also found a high prevalence of moderate to severe malnutrition.

The reported prevalence of pain in this study was 47.0%. Two meta-analyses of studies conducted among patients undergoing hemodialysis estimated a consistent and high prevalence of pain (60% and 60.5%, respectively), which is slightly greater than that reported in the present study^52,53^. Musculoskeletal, peripheral neuropathic, and dialysis-related causes are commonly cited by dialysis patients as perceived causes of pain^49,54^. Due to the high prevalence of pain and its association with mortality and deterioration of kidney function, pain management is key for improving health outcomes during hemodialysis. The responsibility of nephrologists to provide comprehensive dialysis care involves the ability to detect and treat pain while referring to pain specialists in advanced cases only. Therefore, fellowship training programs must integrate pain management into the curriculum, especially since nephrologists receive inadequate training in pain assessment and management^55^.

The prevalence of moderate to severe malnutrition in this study was 45.2%. This finding is in line with the findings of local studies that used the MIS tool, which ranged between 55.9 and 66%^17,32^. Globally, studies have used various tools or classifications to measure the risk of malnutrition but have consistently shown that the prevalence of malnutrition exceeds 50% in most cases^36,56–59^. Malnutrition in patients undergoing hemodialysis can be attributed to iatrogenic factors, such as dialysis-induced nutrient loss and inflammation^60,61^, and/or noniatrogenic factors, such as loss of appetite^62^, taste alteration^63^, and insulin resistance^61^. Furthermore, common comorbid conditions, such as heart failure, may contribute to the worsening of malnutrition in patients undergoing hemodialysis^64^. Given the impact of malnutrition on mortality, psychological state, and cognitive function, optimizing the nutritional status of patients undergoing hemodialysis is the key to improving health outcomes. Therefore, nutritional assessment and management, preferably by a nutritionist, should be an integral part of hemodialysis care.

In addition, malnutrition was significantly correlated with pain in the present study. While no study has investigated the relationship in this patient population, research conducted among other populations has shown similar associations, including among elderly patients^28,65^, patients visiting general practice^66^, and cancer patients living with cancer^67,68^. Another study conducted among a large sample of the public showed that patients with malnutrition are almost 1.5 times more likely to experience chronic pain^69^. However, the directionality and pathophysiology underlying this relationship are unclear. Malnutrition can cause pain through the effects of inflammation, oxidative stress, and the gut-brain axis^30,70^. Pain, on the other hand, can cause depression and affect the cognitive state, leading to loss of appetite and contributing to malnutrition^29,71,72^. The correlation found in the current study is particularly relevant to dialysis patients, as they are a population that has a high prevalence of malnutrition and pain, both of which are associated with considerable morbidity and mortality. Therefore, interventions and training programs should integrate simultaneous management of pain and malnutrition, as these interventions can be mutually beneficial, efficient, and effective.

According to our regression analysis, pain interference with functioning remained associated with the number of comorbidities. This finding is consistent with those of multiple local and global studies conducted among patients who were undergoing dialysis or diagnosed with CKD^49,50,73–75^. The presence of comorbid conditions may cause pain through various mechanisms. Diabetes, atherosclerosis, and bone diseases are common comorbid conditions that can cause pain in patients undergoing dialysis^76–78^. For example, diabetes can cause peripheral neuropathic pain^78^, which is especially relevant in patients diagnosed with CKD, as coexisting uremia can exacerbate neuropathy^79,80^. Furthermore, diabetes is associated with high levels of calcium and phosphate products, which can cause musculoskeletal pain^76^. These findings indicate that comorbid conditions may have synergistic effects on the experience of pain among patients on HD through interrelated mechanisms. The associations of pain with comorbidities and malnutrition further emphasize the need for a comprehensive patient-centered approach to the treatment of CKD in patients undergoing hemodialysis whereby a multidisciplinary team devises management plans customized to the individual patient. These plans should include pain management, nutritional optimization, and integrative treatment of comorbid conditions to reduce the mortality and morbidity associated with hemodialysis.

In addition, the associations of pain with other demographic and socioeconomic factors, including gender and education level, disappeared in the multivariate analysis, except for marital status, which was significantly associated with the pain interference score. These factors represent an interrelated web of environmental factors that are contextual, complex, and variable, especially in different settings. For example, pain in patients undergoing hemodialysis was associated with female gender in a Palestinian study^49^, while it was associated with male gender in a similar study conducted in Taiwan^73^. This adds to the complexity that arises from the influence of gender roles on pain expression and the possible biological and psychological factors that affect pain intensity in males and females^81^.

Additionally, this study revealed that most patients received dialysis for less than four hours per session (91.3%). The duration of the dialysis session is reflective of dialysis adequacy^82–84^. Flythe et al. conducted an observational study that included 10,571 patients and reported that those who underwent dialysis for less than four hours had 26% higher mortality than those who underwent dialysis for more than four hours^85^. Similar to the present study, previous local studies have revealed that the vast majority of patients undergoing dialysis in Palestine received less than 4-h sessions^17,18,86^. This might be attributed to the high costs associated with dialysis in Palestine, which was estimated at an average of 16,085 USD per patient per year^87^. The high cost is fully covered by the government, whose healthcare system faces constant financial crises with resultant shortages in human and physical resources, which explains the reasons behind the short duration of dialysis sessions in pursuit of maintaining services at a lower quality^88,89^.

This study was the first to specifically investigate the correlation between pain and malnutrition in patients undergoing hemodialysis, especially since these two conditions are common and crucial in determining the health outcomes of hemodialysis patients. This study has several limitations, however. First, the lack of temporal assessment inherent in the cross-sectional design precludes the establishment of a causal relationship between pain and malnutrition, which is especially important since the directionality of this relationship remains unclear in the literature. Second, while MIS has been found to be effective, its classification into mild, moderate, and severe has not been successful. The MIS score, a continuous variable that was measured without error, was redefined as a categorical variable whose categories might correspond to inaccurate thresholding of the original continuous values. This could have led to over- or underestimation of the proportion of patients with moderate to severe malnutrition.

## Conclusions

Pain in patients undergoing hemodialysis is associated with mortality and deterioration of kidney function. Malnutrition can also lead to poor health outcomes and increase the odds of mortality in the same patient population. Given that research exploring the pain–nutrition nexus is scarce, this study aimed to explore the potential correlation between pain, on the one hand, and malnutrition and other sociodemographic and clinical factors, on the other, in patients undergoing hemodialysis. The study showed that malnutrition was associated with pain severity. Moreover, pain interference was associated with malnutrition, the number of comorbidities, and marital status. In agreement with most local and global studies, the prevalence of pain and malnutrition was high. To achieve better health outcomes, management plans should adopt an individualized approach that includes pain management, nutritional evaluation, education, and support, with attention given to comorbidities. Furthermore, pain management should be included in nephrology fellowship training to improve nephrologists’ skills in addressing and responding to pain symptomatology.

## Data Availability

The datasets generated and/or analyzed during the current study are not publicly available due to setting restrictions and local Institutional Review Board regulations. However, they are available from the corresponding authors upon reasonable request.

## Abbreviations

LMICs: Low- and middle-income countries
CKD: Chronic kidney disease
GFR: Glomerular filtration rate
NNUH: An-Najah National University Hospital
BMI: Body mass index
WHO: World Health Organization
MIS: Malnutrition-inflammation score
TIPC: Total iron binding capacity
BPI: Brief Pain Inventory
IRB: Institutional Review Board
